# Living with the Late Effects of Childhood Cancer Treatment: A Descriptive Qualitative Study

**DOI:** 10.3390/ijerph18168392

**Published:** 2021-08-08

**Authors:** Hye Chong Hong, Ari Min, Sungkyoung Choi

**Affiliations:** 1 Department of Nursing, Chung-Ang University, Seoul 06974, Korea; julieh@cau.ac.kr (H.C.H.); amin@cau.ac.kr (A.M.); 2 Department of Nursing, Gwangju University, Gwangju 61743, Korea

**Keywords:** childhood cancer, survivors, late effects, qualitative research, life change events, cancer treatment, survivorship, health care, caring science

## Abstract

Long-term childhood cancer survivors (CCS) may experience physical, social, and emotional struggles posttreatment. Our aim was to explore the experiences of CCS dealing with the late effects of cancer treatment from their own perspectives. This study employed a qualitative descriptive design to explore and describe the experience of dealing with late effects among CCS. Semi-structured interviews were conducted with 15 CCS in Korea. Participants were selected by purposive and snowball sampling and individually interviewed during the period from September to November 2020. Conventional content analysis was used to analyze data and identify themes. Two main themes and seven subthemes emerged. The two main themes were: “Things I encountered while crossing a bridge” and “Living as a survivor”. The participants reported both positive and negative experiences with dealing with the late effects of cancer treatment. The main themes indicated that late effects exert significant impacts on the lives of CCS in both positive and negative ways. Healthcare providers and researchers should pay attention to early intervention needs of CCS and their support systems to strengthen their positive experiences in dealing with late effects during their survivorships.

## 1. Introduction

With the advanced and aggressive treatments used in childhood cancer, the 5-year survival rate for childhood cancer is over 80% in the United States and European countries [1,2]. Similarly, the current 5-year survival rate for childhood cancer in Korea is about 84%, which is a dramatic increase from 56% in 1995 [3,4]. While the steady increase in the number of childhood cancer survivors (CCS) has focused the attention of researchers on survivorship periods, there is still a lack of research on CCS’ experiences during their survivorship.

Long-term CCS may experience physical, social, and emotional struggles post treatment [5]. Unfortunately, approximately 20% of CCS have at least one moderate late effect, and more than 10% have disabling life-threatening late effects [6]. Late effects are health problems or complications of cancer treatments that affect survivors physically and psychologically; these effects can develop months or years after the completion of cancer therapy [7]. Such late effects include cardiovascular, metabolic, skeletal, reproductive, and neurocognitive symptoms and disorders [6,8,9]. CCS must be counseled and continuously screened for long-term late effects of their specific therapies; however, many tend to be unaware of the potential late effects of cancer treatment during childhood, which could result in uncertainties such as risk of relapse and anxieties about life and death [10,11,12]. Duffey-Lind et al. [13] reported that young adult cancer survivors perceived a lack of support and information while transitioning off their cancer treatments. Other barriers to successful adjustments to post-cancer treatment include the difficult and abrupt transition from pediatric to adult health services, inconvenient and under-resourced health services, family doctors’ inadequate experience with late effects management, and overdue and inadequate late effects communication with CCS [14]. Moreover, Hendriks et al. [15] explored unmet needs of CCS in long-term follow-up care and reported lack of psychosocial support, lack of collaboration and decentralization of care, and the need for centralized and individualized services for CCS.

Importantly, CCS face significant challenges in adapting to society due to the late effects of intensive treatment during childhood. Further, these late effects can lead to difficulties in adjusting to life stages including education, marriage, childbirth, and employment [16,17,18]. Because the treatment during childhood can also affect growth, and cause reproductive and sexual development problems, the CCS’ perceptions of their bodies could lead to emotional challenges, low self-esteem, poor quality of life, and difficulties in social functioning [19,20,21]. However, experiencing childhood cancer is not entirely negative. In fact, the trauma of experiencing childhood cancer can promote growth in CCS. Life-threatening illnesses may provide positive outcomes such as prosperity, self-renewal, resilience, and achievement of personal and emotional growth that enhance quality of life [22,23,24].

However, there is limited research on childhood cancer and the experience of late effects during survivorship, especially in Korea. In our prior quantitative research, it was found that more than 50% of CCS in Korea experience late effects of cancer treatment and that there is a need for tailored interventions for CCS with different numbers and intensities of late effects [25]. Yet, this prior research was fractional and used generic quantitative measures, which limited the ability of CCS to report real experiences that are significant to them [26]. CCS’ voices are scarcely found in literature related to the real experiences of late effects, how these impact on daily life, and what CCS can do to cope with them. Hence, this paper aims to explore CCS’ experiences of dealing with the late effects of cancer treatment.

## 2. Materials and Methods

### 2.1. Aims

This paper aims to explore CCS’ experiences of dealing with the late effects of cancer treatment.

### 2.2. Design

A qualitative descriptive design was employed to explore and describe the experience of dealing with late effects among CCS. This design is appropriate because we aim to explore naturalistic perspectives of the late effects of CCS in their natural states. Moreover, a qualitative description provides flexible commitment to a theory or framework and allows insightful analyses of phenomena and experiences [27,28,29]. We have used Sandelowski’s guidance of a qualitative descriptive study [27,28] and applied the Consolidated Criteria for Reporting Qualitative Research (COREQ) checklist to report the findings [30].

### 2.3. Sample/Participants

We applied purposive and snowball sampling to source CCS participants. Eligible participants: (a) were diagnosed with childhood cancer at an age younger than 19 years; (b) received treatments including chemotherapy, radiation therapy, surgery, or hematopoietic stem cell transplantation; (c) are currently aged between 19 and 39 years; and (d) are at least two years after their cancer treatment completion. First, we posted online advertisement on the webpages of CCS self-help groups and Korean Association for Children with Leukemia and Cancer (KACLC) to recruit participants. Second, participants were encouraged to send the advertisement to other known CCS. Participants who met the eligibility criteria and were interested in participating provided their contact information through a registration link. A total of 18 CCS registered. The first author contacted participants to provide information regarding the study’s purpose and interview procedures and reconfirmed whether they met the selection criteria. Three participants were excluded from the study due to their current age being less than 19 years (1 case), experiencing no existing late effects (1 case), and not being diagnosed with childhood cancer (1 case). Finally, 15 CCS participated in the individual semi-structured interviews. 

Data collection (interviews) began after obtaining approval from the Institutional Review Board (IRB) at a university (no. 1041078-202004-HR-101-01). Participants were informed of the study purpose and procedures. Signed informed consent was obtained voluntarily from each participant prior to the interview. Participants were also notified that they had the right to withdraw at any time.

### 2.4. Data Collection

Qualitative interview data were collected from September to November 2020. Semi-structured interviews took place in a private meeting room either in-person or via Zoom, an online videoconferencing program. The online interviews were used to overcome data collection challenges and provide flexibility of timing and location for the interviews due to the COVID-19 situation.

All individual interviews were conducted by the second author, who has received training and is experienced in qualitative research methods. The interviewer had experience in working with childhood cancer patients in pediatric units and was thus suitable to build trust with participants. Interviews were conducted according to a semi-structured interview guide developed through a literature review and research team discussion (refer to the Supplementary Table S1). The guided interview consisted of an introduction to break the ice; interview questions on experiences of late effects; member checking, if necessary; a summary; and closing remarks. The interviewer used open-ended questions and targeted probes to guide participants, allowing them to speak freely about their experiences of the late effects. Participants were encouraged to recall and describe specific experiences related to their coping and management strategies for the late effects, including the challenges with and opportunities gained from these strategies. As the interview progressed, redundant probes were removed, and new questions based on emerging themes in the initial interviews were added. Interviews lasted between 60 and 90 min and were conducted once per participant. All interviews were digitally recorded and subsequently transcribed verbatim by a research assistant. The first and second authors reviewed the transcripts against the recorded audio files to ensure accuracy and remove participants’ identifiers. Interviews were completed and analyzed simultaneously to inform following interviews. Interviews continued until saturation was achieved when no new themes emerged.

### 2.5. Data Analysis

**Figure 1:**
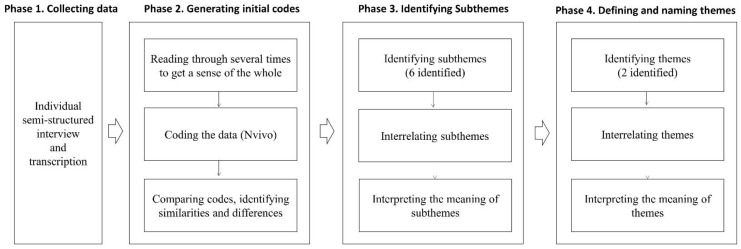
Overview of the analysis process in the study.

Conventional content analysis was used to analyze interview transcripts and identify themes [31] (Figure 1). The first and second authors individually read the transcripts several times to familiarize themselves with the data, then coded significant words and phrases independently. In the case of disagreements between the two authors, the research team had a debate process to reach consensus and confirm the final codes. Subsequently, codes were examined to identify commonalities and grouped together as subthemes. The subthemes were further abstracted to identify the key concepts related to CCS’ experiences of late effects. The whole process from reviewing the codes to generating subthemes and themes was carried out by the full research team to discuss and to reach consensus. The words used by CCS were reported verbatim to retain their original meanings throughout the analysis and quotes used in this manuscript were selected by consensus during meetings. Data were analyzed and managed using the NVIVO software, version 13 (QSR International, Warrington, UK).

### 2.6. Rigor/Trustworthiness

To establish the trustworthiness of the study, we used investigator triangulation, member checking, peer debriefing, researcher reflexivity, and audit trailing [32,33]. The research team consisted of three nursing professors who had experience working with childhood cancer patients in pediatric units and specialized in CCS research and the methodological field of qualitative research. All the steps in coding and data analysis for theme abstraction were conducted by research team members independently, and then confirmed through communication to discuss and agree upon the results of the data analysis. In addition, member checks were frequently conducted during interviews. The interviewer summarized what participants said and asked participants to verify the interviewer’s interpretations. In peer debriefing, an external peer (a nursing expert in qualitative research) reviewed the data and research process and interrogated the procedures, meanings, interpretations, and conclusions of the investigation. An audit trail was maintained by the researchers for the entire research process. Raw data, interview notes, coding rationale, and research team meeting minutes were recorded in detail and kept in password protected files. In addition, the research team members discussed their prior knowledge and preconception about the study before the study commenced and maintained reflective journals throughout the research process.

## 3. Results

A summary of participants’ demographic characteristics is provided in Table 1. Of the 15 participants, six were male and nine were female. The average age was 22.8 years (range = 19–26 years) and all were unmarried. Three participants were Christian, three were Catholic, and the rest did not have a religion. Most participants were undergraduate students (10 participants), and three participants were currently employed. Nine were diagnosed with leukemia, four with malignant lymphoma, and two with osteosarcoma. Their ages at the time of diagnosis ranged from 2 to 18 years old, and their ages when the treatment ended ranged from 7 to 20 years old. All participants were treated with chemotherapy; among them, five participants also received radiation therapy and hematopoietic stem cell transplantation. Three participants experienced relapses.

Two main themes emerged from the content analysis: (1) Things I encountered while crossing a bridge, and (2) Living as a survivor. The themes and subthemes are presented in Table 2.

### 3.1. Theme 1: Things I Encountered While Crossing a Bridge

CCS who were discharged after cancer treatment were left to face various late effects alone and often struggled to handle these late effects. The first theme, “Things I encountered while crossing a bridge”, which refers to the experiences of coping with late effects among CCS, is classified into four subthemes: (1) Do not know where to get it, (2) Not helpful at all, (3) Worsening my situation, and (4) People were there when I needed them.

#### 3.1.1. Do Not Know Where to Get It

The CCS felt lost as they did not know what to do and whom to ask when they experienced late effects. They expressed feeling “frustrated,” “baffled,” and “lost.” Additionally, when they experienced relatively mild physical symptoms such as dizziness, difficulty concentrating, and lack of sleep, they were confused about whether these symptoms were sequelae of their cancer treatment or also experienced by those who have not undergone cancer treatment. Participants sought solutions by searching the Internet; however, they were often unsuccessful in finding answers. They searched for information on the same symptoms, specifically for CCS, and information related to the experiences of other CCS. However, no information was available.

I just didn’t know what to do when I got sick. I wouldn’t know where to go. I searched on the internet a lot, but … there are only advertisements and not what I am looking for.… I felt somewhat frustrated about that.

(Participant 10)

#### 3.1.2. Not Helpful at All

The CCS most often asked health care providers for help when they experienced late effects; however, most of the participants stated that the health care providers were unable to provide adequate assistance. Participants noted that they did not receive sufficient explanation from the health care providers regarding the late effects that may occur after treatment. Participants mentioned the importance of discharge education about the late effects upon the completion of cancer treatment so that they are aware of symptoms and prepared for their occurrence. In addition, although the CCS had regular follow-ups after treatment, the appointment durations were too short, and the health care providers were often so busy that the CCS could not discuss their late effects. When CCS consulted their doctors about their late effects, doctors were unhelpful and only responded with: “It is ok. That is normal,” and “You just have to live with it.” Participants often received treatment from departments other than oncology due to late effects such as infertility, decreased renal function, hormone imbalance, and hip dysplasia. The CCS expressed that doctors often lacked specialization in childhood cancer and, thus, had limited understanding of late effects and the best strategies for treatments thereof.

When I get late effects, I can’t go to a general clinic because I am not a general patient … those doctors in general clinics are not specialized in pediatric cancer, so … there are a lot of limitations because they treat me like one of the general patients.

(Participant 7)

#### 3.1.3. Worsening My Situation

Certain situations were beyond being unhelpful and negatively affected the physical, psychological, and social aspects of CCS’ lives. High-dose chemotherapy or radiation therapy are highly likely to cause infertility; however, none of the CCS were not notified of this by health care providers prior to the treatment process. Thus, the CCS were not provided opportunities to store sperm or eggs, and were thus deprived of their wish to have biological children.

I initially tried to store sperm before hematopoietic stem cells transplantation. But they told me there was no sperm. (Interviewer: Before the transplant?) Yes, because I had chemotherapy before…. The health care providers did not tell me this before using chemotherapy … there was no explanation at all … they don’t tell you. Because these people [health care providers] think that the faster the treatment begins, the better, and do not consider … they just want to start treatment right away.

(Participant 3)

In terms of psychosocial aspects, the worries of their parents often led to overprotection, hindering the CCS from developing self-identities and becoming independent adults. In school, some of the CCS were forcibly asked to remove their hats, which they wore to cover their hair loss, and participate in physical activities without exception, resulting in psychological and social atrophy.

My mother was extremely overprotective… She only focused on the fact that I was sick, and she tried to lock me in. So, there was often a conflict of opinions between me and her, and she kept on saying, “you need to be careful because you were sick when you were a child, you cannot drink,” always giving me more rules, and I always wanted to be just another college student like everyone else and wondered why I could not be one. So, I understand that I got treatment when I was younger, but why does she keep saying no? … There were some conflicts then … I never told my mother, but I hated how she overprotected me as a childhood cancer patient, and I think it took me a while to get out of it myself.

(Participant 10)

#### 3.1.4. People Were There When I Needed Them

Some CCS acknowledged that they were responding to late effects effectively with the help of health care providers, family members, self-help groups, and social support. Participants stated that some health care providers heeded their rights as survivors by explaining to them beforehand about the late effects that may occur after cancer treatment. Participants described that their parents played a key role in encouraging and guiding them—as they were disconnected from society during treatment—to move forward and adapt to the changing world. The CCS were also able to communicate with and receive emotional comfort from other CCS by sharing their concerns and gaining knowledge on late effects through self-help group meetings. CCS meetings were held through private gatherings, systematically organized by doctors and hospitals, or the non-profit organization, KACLC. The CCS also participated in social adaptation training or counseling programs organized by the KACLC.

Participant 8 stated, “In my case…there are self-help groups at the hospital. Gatherings of sick people. I go there to share my experience and to gain comfort … and I talked a lot with my mother and father, received a lot of counseling.”

### 3.2. Theme 2: Living as a Survivor

The CCS who struggled to manage the late effects of childhood cancer treatment were living as survivors in their own ways, carrying enduring memories. The second theme, “Living as a survivor,” focuses on the current lives of CCS living with late effects. This theme is classified into three subthemes: (1) Enduring and accepting, (2) Found my own coping strategies, and (3) My life is special.

#### 3.2.1. Enduring and Accepting

When symptoms of the late effects did not improve, the CCS accepted their situations of despair, enduring the pain rather than actively searching for solutions. These symptoms included decreased physical strength, loss of nails, slow gait due to hip joint problems, hair loss, and infertility. The CCS consoled themselves with the reasoning that they had no choice but to live with the symptoms of their past chemotherapy that used toxic anti-cancer drugs. CCS stated that when they were overwhelmed with fear of their cancer’s recurrence, they avoided and ignored negative thoughts by telling themselves that, “it would not happen again” (Participants 4, 7, 10). One CCS reported that they drank alcohol and exercised in their daily lives like any other healthy person because they did not want to live a life full of anxiety about the possible recurrence of cancer. The participants acknowledged that they became used to the late effects over time and reported that the late effects were no longer an inconvenience to their daily lives.

When I visit the obstetric clinic, I always hear the same thing, you know: you can’t have babies. So, emotionally it gets to me even though I know that fact … in my case, I love children so much … my mother and father try to encourage me. They think I can have babies because future technology may allow this … but I am a more pragmatic person and … tend not to think about far into the future. I don’t expect much.

(Participant 8)

#### 3.2.2. Found My Own Coping Strategies

Some CCS created their own methods to cope with their late effects. When they had headaches, they took painkillers, and when gastrointestinal symptoms appeared, they limited their intake of spicy foods to control diarrhea. The CCS also showed great understanding of their physical strength levels and found an appropriate balance between activities and rest to live their best daily lives in their given circumstances. They went home when necessary for rest and replenished their energy when they felt tired. They also regularly consumed nutritional supplements and exercised to improve their stamina. The CCS found their own ways to complete their given work without overwhelming themselves. For example, participants allocated time for work and rest each day and created checklists. When symptoms of suspected recurrence appeared or extreme fear overwhelmed them, they visited their nearest hospital for blood tests to relieve their anxiety.

I am a perfectionist, and I keep working on something until it is of the level that I find satisfactory. I cannot sit for long because I don’t have any muscles in my buttocks because of Estradiol injection. So, I divide time to finish my assignment. I assign time … from what time to what time, I finish this assignment and then have a 30-minute rest and then come back to do the assignment. I ensure that I go to bed by a certain time to have strength for the next day.

(Participant 8)

#### 3.2.3. My Life Is Special

All the CCS participants expressed that they were grateful to be alive after going through cancer treatment. Furthermore, they were proud of themselves for enduring the painful treatment process after being diagnosed with childhood cancer, and positively accepted their lives and reported spiritual growth. These thought processes were often initiated by religious beliefs and coincidently encountered mentors. They were also motivated by other parents who had children with childhood cancer. One participant stated, “this life itself is special” (Participant 1). The participants not only considered their lives special, but also shared their dream of helping sick and troubled individuals. The CCS formed a volunteer group for children with cancer and their parents. Those studying and those who had completed their studies chose majors such as social welfare or counseling psychology, and one participant worked as a social worker at the Korea Pediatric Cancer Foundation, positively sublimating their cancer treatment experience.

By chance, I started volunteering at the Korean Association for Children with Leukemia and Cancer…. While volunteering, I met a good mentor, and the mentor told me that I was brave after hearing my history of sickness. People always told me, “you are different [from all these healthy people],” and then I met someone who changed my perspective by saying, “it’s so great that you overcame this”.… Since then, I came to accept the fact that I was sick [positively]…. The reason I chose social welfare studies was because I wanted to work for people with childhood cancer. I was once a person who received support and wanted to give back to society, so I graduated with a degree in social welfare and worked with the Social Work Team at the Seoul St. Mary’s Hospital last year.

(Participant 10)

## 4. Discussion

Our participants were relatively young and were preparing to play active roles in society at large. Their health (late effects) is particularly important for these economically active CCS as late effects may lead to difficulties in adjusting to social roles such as education, marriage, childbirth, and employment [16,17,18]. In contrast to adult cancer survivors, CCS in young adulthood may encounter more negative experiences due to the transitional period of life and their limited experience with life. However, consistent with previous research, our participants reported both positive and negative experiences in dealing with the late effects of cancer treatment [34,35]. One negative experience found both in previous research and this study is the lack of explanation about the late effects of cancer treatment (refer to subtheme “Do not know where to get it”). These late effects ranged from mild physical and psychological symptoms to infertility in later life. CCS were frustrated and perplexed when they experienced late effects because they were not aware that these symptoms could be due to their cancer treatments and did not know where to seek help for further affected physical, psychological, and social aspects of their lives.

One significant late effect mentioned by several CCS was infertility. They were not notified of the possibility of infertility prior to their treatment processes even though CCS have been found to have a 50% increased risk of infertility, compared to healthy controls [36]. In the United States, the American Society for Clinical Oncology recommends that healthcare providers discuss the risks of infertility and provide information about fertility preservation [37]. It is known that understanding one’s own disease and treatment is closely related to a higher quality of life [38] and receiving specific counseling about potential reproductive loss and fertility preservation is associated with less regret and higher quality of life for cancer survivors [39]. However, no guideline is available in Korea to discuss the late effects of childhood cancer treatment. Moreover, discussion about late effects, such as infertility, may not be a priority for healthcare providers during active and emotionally challenging treatment periods. Nevertheless, our participants indicated the need for early information about late effects during treatment periods or follow-up appointments (refer to subtheme “Not helpful at all”). In the Korean healthcare system, oncologists or hematologists are the first point of contact during active and follow-up periods. It is, therefore, important that these healthcare providers are trained about late effects and how and when to communicate relevant information about them. The experience of late effects may differ for each childhood cancer survivor. The development of tailored survivorship care plans that could monitor and inform survivors and healthcare providers about possible late effects and long-term effects of cancer treatment, as well as provide instructions for follow-up care that includes the management of late effects, are necessary for CCS in Korea.

Diagnoses of childhood cancer and the treatment thereof are also traumatic events for parents of CCS. A child’s serious illness may be a challenging for parents and the perception of their child’s vulnerability to a cancer relapse may impede the healthy development of a child–parent relationship [40,41]. Parental stress post-cancer treatment of the child has been associated with high levels of anxiety, depression, fear of recurrence, worry, and fatigue [42,43,44], and may further lead to parental overprotection [45]. Research found that family and parental factors play important roles in the health outcomes of CCS; parental distress was associated with overprotective behavior and interfered with the quality of life of CCS [46]. In fact, the overprotection due to the worry of parents was found to have led to conflicts between CCS and parents in our study (refer to subtheme “Worsening my situation”). Overprotection may have a negative influence on CCS achieving their independence and autonomy [46]. In addition, parental overprotection may also lead to a reduction in time spent with peers and social isolation [47]. However, parental distress and the psychological states of parents of CCS is underestimated and rarely studied in Korea. Qualitative approaches to explore the parents’ distress and psychosocial needs, describe the relationship between parents and CCS, and explore the mechanisms between parental distress and overprotective behaviors during treatment, as well as survivorship periods, are needed. We also recommend developing early monitoring and counseling for both CCS and their parents to identify problems and early interventions to build healthy relationships.

Cancer survivors might be discriminated against and rejected in society because of the stigma attached to cancer and cancer survivorship [48]. Cancer survivors are prone to public stigma because cancer treatment may lead to visible physical and developmental outcomes such as hair loss, scarring, and a small frame [49,50,51,52]. Researchers reported that public stigma is associated with depression and subjective wellbeing in cancer patients [53,54,55]. Public stigma is particularly important for CCS as many returned to school after periods of social isolation due to cancer treatment. Childhood cancer treatment may influence the social functioning and peer relationships of CCS as they are seen by their peers as sick, fatigued, and absent from school [56,57]. Ignorance of teachers and peers may add to the stress experienced by CCS when returning to school [58], and abuse and taunting from their peers were reported due to changes in the physical appearance of CCS; this can further lead to a low self-esteem, reduced social interactions, and self-induced isolation of CCS [59]. Public education is vital to reduce the stigma of childhood cancer and CCS, but it may show delayed effect. Meanwhile, it would be helpful to provide supportive environments to CCS, such as support groups with other CCS who can share their positive experiences with social adjustment [48]. Social support is known to be a protective factor against stress and, therefore, important in the lives of cancer patients [48]. In our study, CCS reported that they received emotional comfort from other CCS (refer to subtheme “People were there when I needed them”). For young survivors, peer support groups can be beneficial because they allow CCS the opportunity to share similar experiences and their coping strategies, especially when many Koreans do not feel comfortable disclosing their cancer diagnosis [60]. Yet, there is a limited number of peer support groups available in Korea and we believe more should be organized by hospitals that have close contacts with CCS and non-profit organizations. We also recommend that future research related to specific factors leading to peer difficulties and social isolation should be conducted to develop further interventions or programs for CCS. Public education about cancer and its late effects is crucial; however, it would take some time to have an effect. An educational program and campaigns for the public, including schoolteachers, about cancer and its late effects would be a good start and beneficial in the future.

One interesting finding of our study was that some CCS positively sublimated their experiences with cancer and treatment and dreamed of helping sick childhood cancer patients and forming and participating in volunteer groups for children with cancer and their parents (refer to subtheme “My life is special”). CCS who sublimated their experiences talked about their spiritual growth during and after cancer treatment. Survivors of traumatic events such as being diagnosed with cancer and going through painful treatment processes try to understand the stressor, and spirituality plays an important role in coping with such events [61,62]. In our previous research, we have also found a positive relationship between spiritual growth and quality of life among CCS [63]. Our participants reported that they were positively influenced by mentors and parents who had children with childhood cancer during their survivorship periods, which indicates the importance of providing support after cancer treatment completion. However, we know little about the role of spirituality among CCS and which factors influence spiritual growth and positive sublimation, especially in Korea; therefore, we recommend further quantitative research and the future development of intervention programs based on the factors associated with spiritual growth and positive sublimation.

A major difference between the present and previous studies is our analysis and findings of both positive and negative experience with late effects from the perspectives of CCS; this is also a strength of this study. These findings provide new knowledge about dealing with late effects among CCS, especially in Korea, where research into CCS is still in its infancy. However, some limitations should also be noted. Selection bias could be present as we recruited the majority of participants from the KACLC. Although we tried to recruit a more varied pool of participants using the snowballing technique, the majority of participants had leukemia or lymphoma. Recruiting CCS in Korea is often difficult, as many CCS may be reluctant to reveal their medical/cancer history. Moreover, because the participants were in their 20s, the experiences of older survivors need to be explored further. We recommend that studies with larger samples, including various cancer diagnoses and age groups, be conducted in the future to confirm our study findings.

## 5. Conclusions

The main themes found in our study indicated that late effects exert significant impacts on the lives of CCS in both positive and negative ways. Unlike adult cancer survivors, CCS in young adulthood may endure more negative experiences due to their limited capability and exposure to life, and limited support from health care providers, peers, and families. Our study findings provide a basis for future studies in several ways: First, it is necessary to develop tailored survivorship care plans that can monitor and inform both CCS and health care providers about the late effects of childhood cancer treatment. Second, developing counseling programs for both CCS and their families would be useful for building healthy relationship and socialization. Lastly, researchers need to develop interventions that could reinforce the positive experiences associated with late effects, such as spiritual growth and outreach. We also recommend that future studies include more diverse CCS in terms of age and cancer diagnoses to fully understand the experience of late effects in CCS.

## Figures and Tables

**Table 1:** Participants’ demographic characteristics.

	Gender	Diagnosed Age (years)	Treatment end Age (years)	Current Age (years)	Diagnosis	Recurrence	Type of Treatment			Religion	Education Level	Current Job
							Chemotherapy	Radiation Therapy	Hematopoietic Stem Cell Transplantation			
1	Male	2	7	25	Leukemia	No	Yes	No	No	Christian	Baccalaureate	None (seeking a new job)
2	Female	12	14	20	Leukemia	Yes	Yes	Yes	Yes	None	High school	Undergraduate student
3	Male	18	20	25	Malignant lymphoma	No	Yes	No	Yes	None	High school	Part time job
4	Female	14	15	25	Malignant lymphoma	No	Yes	No	No	None	High school	Undergraduate student, Part time job
5	Male	12	18	25	Leukemia	No	Yes	No	No	None	Baccalaureate	Coffee shop manager
6	Female	16	16	26	Malignant lymphoma	No	Yes	No	No	None	Baccalaureate	Graduate student
7	Female	14	17	20	Osteosarcoma	Yes (2 times)	Yes	No	Yes	Catholic	High school	Undergraduate student
8	Female	13	13	20	Leukemia	No	Yes	Yes	Yes	Christian	High school	Undergraduate student
9	Male	15	16	23	Osteosarcoma	No	Yes	No	No	Catholic	High school	Undergraduate student
10	Female	5	8	25	Leukemia	No	Yes	No	No	None	Baccalaureate	Social worker
11	Male	18	19	22	Leukemia	No	Yes	No	No	None	High school	Undergraduate student
12	Female	13	13	19	Leukemia	No	Yes	No	Yes	None	High school	Undergraduate student
13	Male	14	20	23	Leukemia	No	Yes	Yes	Yes	Christian	High school	Undergraduate student
14	Female	13	18	25	Leukemia	Yes	Yes	Yes	Yes	Catholic	High school	Undergraduate student
15	Female	13	15	19	Malignant lymphoma	No	Yes	Yes	Yes	None	High school	Undergraduate student

**Table 2:** Main themes and subthemes.

Themes	Subthemes
Things I encountered while crossing a bridge	Do not know where to get it
	Not helpful at all
	Worsening my situation
	People were there when I needed them
Living as a survivor	Enduring and accepting
	Found my own coping strategies
	My life is special

## Data Availability

To protect the confidentiality of the research participants, the data are not made publicly available.

## Supplementary Materials

The following are available online at https://www.mdpi.com/article/10.3390/ijerph18168392/s1, Table S1. Individual in-depth interview guide.
