# Supplementary material for: Living with the Late Effects of Childhood Cancer Treatment: A Descriptive Qualitative Study
Source: Int J Environ Res Public Health. 2021 Aug 8;18(16):8392. doi: 10.3390/ijerph18168392 (PMC8393717; doi:10.3390/ijerph18168392)
Supplement: Supplementary file 1 [file ijerph-18-08392-s001.zip › ijerph-1273488-supplementary.pdf]

Supplementary Table S1. Individual in-depth interview guide

Searching point	Key questions
Introduction	- Welcome and introduction of researchers<br>- Explaining the interview process and confidentiality
Experience with late effect	- When and how did the late effect appear?<br>- Do you have any particularly memorable experiences with the late effect?
Experience with symptom management of late effect	- What did you do to manage the symptoms of late effect and how did it/they affect you?<br>- What helped or hindered you in managing your late effect symptoms?<br>- Do you have any memorable experiences related to symptom management?
Additional questions	- What does living with the late effect mean to you?<br>- Is there anything else you would like to add?
Summary and closing	- Summarize the interview
